# CULTIVATING TRUST IN RESEARCH PARTNERSHIPS WITH OLDER ADULTS ACROSS THE COMMUNITY ENGAGEMENT CONTINUUM

**DOI:** 10.1093/geroni/igae098.2015

**Published:** 2024-12-31

**Authors:** Allyson Brothers, LIza Behrens, Carrie Leach

**Affiliations:** Chair; Co-Chair; Discussant

## Abstract

Community-engaged research is essential for identifying and supporting the needs of an aging society and ensuring effective translation of research findings. While not a new idea to the field of gerontology, engaged research models and frameworks are expanding, and researchers are seeing increased access to initiatives for building and sustaining community-engaged research programs. This symposium will feature emerging and established projects across multiple levels of the continuum of engagement, from informing, consulting, involving, collaborating, and ultimately to co-creating. Kim et al. will describe efforts to co-create programming and educational materials with and for Alaska Native caregivers, focusing on decolonizing methodologies. Halvorsen will showcase an ongoing academic-organizational partnership created to increase the research and evaluation capacity of a national nonprofit organization and its grantees. Gan will describe how diverse community members were engaged to understand and meet gaps in cognitive health promotion, resulting in the pilot of a mindful discussion program. Guest et al. will present on the formation and fruitful efforts of a resident advisory board at the University Based Retirement Community, sharing best practices for sustaining engagement. Mahmood et al. will report on a community-based participatory effort evaluating the role of built environment on mobility access across five cities in the Vancouver area. These exemplars will launch the symposium into a discussion about the utility of engaging older adults across the continuum, illustrating effective strategies for establishing trusted academic-community partnerships. We invite attendees to consider the value of this heuristic framework to plan and design fit-for-purpose community-engaged research projects. Community Engaged Research Interest Group Sponsored Symposium

